# Psychological symptoms in women in a primary care setting in Tamil Nadu

**DOI:** 10.4103/0019-5545.43060

**Published:** 2005

**Authors:** Edwina Lawson, Tom Craig, Dinesh Bhugra

**Affiliations:** * Honorary Research Worker, Health Services Research Department, Institute of Psychiatry, King's College, London; ** Professor, Health Services Research Department, Institute of Psychiatry, King's College, London; *** Professor, Health Services Research Department, Institute of Psychiatry, King's College, London

**Keywords:** Psychological symptoms, common mental disorders, Self-Report Questionnaire (SRQ)

## Abstract

**Background:**

Common mental disorders, especially depression, are likely to increase as a result of globalization and industrialization and it is likely that the resultant burden of care will increase proportionately. Women have a higher prevalence of depression and also carry the burden of caring for the affected individuals.

**Aim:**

To study the psychological symptoms with possible common mental disorders in a primary care setting.

**Methods:**

One hundred two women of Tamil ethnicity were approached to take part in answering the Self-Report Questionnaire (SRQ). The mean age of cases and non-cases were 39 years and 33 years, respectively.

**Results:**

Nearly three-fifths scored above the cut-off point. Age, physical illness and chronic pain were found to be important factors in the genesis of depression in particular.

**Conclusion:**

These findings have major implications for any preventative or intervention strategies.

## INTRODUCTION

Common mental disorders place a tremendous burden on sufferers and their families. The prevalence of common mental disorders varies from 14.6% to 28% depending on a number of factors.^1^,^2^ Various studies from developing countries indicate that low socioeconomic status, poor educational attainment, poor marital relationship, unemployment and overcrowding all play a part in the aetiology.^3^

Chowdhury *et al.*^4^ reported that in a primary health centre and satellite clinics, 12.6% of patients had major depressive disorder. In a community-based setting, Prasad *et al.*^5^ using the GHQ-12 in 622 women, found that nearly a quarter had reproductive tract complaints indicating physical symptom presentation, and they concluded that the risk of common mental disorders increased with physical symptoms of the reproductive tract. Pothen *et al.*^6^ found a 33.9% prevalence of common mental disorders in subjects attending a primary care centre. In Tamil Nadu, Nambi *et al.*^7^ found that unexplained somatic symptoms were associated with common mental disorders, and a majority of patients held very strong beliefs that their symptoms were caused by physical disorders. Epidemiological studies often use different screening instruments and population samples.

In the Indian subcontinent, the prevalence of somatization is said to be higher, where patients with anxiety and depression present with bodily symptoms rather than pure psychological symptoms.^2^,^3^ The diagnosis of common mental disorders depends upon a number of factors such as where the sample is collected from and what assessment instruments are used.

The present study aimed to ascertain the frequency of psychological symptoms and possible common mental disorders in a rural population in Tamil Nadu. As the rates of common mental disorders are higher in women it was decided to focus on this group.

## METHODS

### Sample

The sample was collected from four villages. The subjects were approached using a purposive sampling technique by the health care workers attached to the community health centre which served these four villages. The subjects knew the community workers and verbal consent was obtained for research. The subjects were assured that refusal to participate would not affect their immediate or future care. Those <17 years of age were excluded. Subjects were selected from a number of settings.

### Location

The first author was based in a primary health care centre (100 km from Chennai) which provided health care services to these four villages. Most of the population worked as casual labourers on a daily wage. The rural economy is agrarian and a majority of the population is under 45 years of age and illiterate. The houses are small and there is overcrowding (most families have between three and five children). The base population of the villages was variable due to a large number of individuals travelling elsewhere regularly.

### Assessment

It was decided to use the Self-Report Questionnaire (SRQ)^8^ for assessment of psychological symptoms and possible caseness. This has been used extensively in a number of cultural settings. A Tamil translation of the abridged 20-item scale was used. Each statement relates to a single symptom and requires a simple Yes/No answer. The SRQ-20 is quick and easy to use (by health workers) and to answer (by subjects) and is therefore appropriate in primary health care settings. Five health workers were trained in the use of SRQ-20 by the first author [EL] and the meaning of each statement was discussed and agreement obtained. No formal tests of interrater reliability were carried out. Basic sociodemographic data were collected on each subject who agreed to participate in the study.

### Analysis

Data were analysed using the SPSS (version 11.00). Univariate associations were explored using statistics as appropriate. Multivariate association was undertaken using logistic regression.

## RESULTS

The sample comprised 102 women. Over 70% of the sample was less than 44 years of age; a majority of them were 25–34 years of age.

Of the 102 participants, 61% had an SRQ score ≥9, and were therefore suspected cases of depression. Univariate factors associated with suspected cases of depressive and anxiety disorder (SRQ ≥9) are presented in Tables 1 and 2.

**Table 1:** Univariate factors associated with suspected cases of depressive and anxiety disorder (SRQ ≥9; chi-square test)

Risk factors for possible caseness (SRQ score ≥9)	Case *n* (%)	Non-case *n* (%)	P	Odds ratio (95% CI)
Physical illness or pain	66 (45)	30 (10)	0.001	4.5 (1.8–11)
On medication	34 (23)	12 (4)	0.021	3.77 (1.2–11.8)
Victim of domestic violence	62 (43)	36 (12)	0.014	2.9 (1.2–6.8)
Alcohol consumption by husband	42 (29)	39 (13)	0.549	1.1 (0.48–2.6)
Two meals a day (rather than three)	36 (25)	3 (1)	0.001	16.4 (2.1–125)
Lack of recent income (<1month)	78 (54)	53 (17)	0.018	3.2 (1.3–7.8)
≥2 children	76 (70)	71 (34)	0.044	2.8 (1–7.6)
Widow	20 (14)	9 (3)	0.156	2.5 (0.78–8.1)
Family death or illness (adverse life events)	32 (27)	41 (22)	0.327	0.7 (0.34–1.4)

**Table 2:** Proportion of possible cases by age group (chi-square test)

Age groups (years)	Case (%)	Non-case (%)
18–24 years	37	63
25–34	56	44
35–44	64	36
45–54	76	24
≥55	77	23

(p=0.049)

The mean age of cases was 39 years, compared with 33 years for non-cases (Mann–Whitney Z= −2.842, p=0.004) and when age was categorized by decade, the percentage of cases increased with each decade (χ^2^=9.541, df=4, p=0.049) (Table 2). Women with 2 or more children were at higher risk of being cases (OR=2.8, CI=1–7.6). Widows did not have a significantly higher case percentage (p=0.156).

Participants who were coping on two meals a day, as compared to three, were at much higher risk of being cases (p=0.001). However, caseness was not predicted by the duration of unemployment and monthly income. This may be due to the homogeneous socioeconomic characteristics of the population. Of the sample, 75% earned very small wages from farming, and 95% reported being unemployed for up to 2 years due to drought.

Only a very small proportion of women and/or their husbands had regular and secure employment (e.g. as bus drivers, or office workers). When this minority of professionals was compared with the majority of poor, unemployed farmers, there was an association between the lack of recent income and caseness (OR=3.17, CI=1.3–7.8, p=0.018). Other markers of socioeconomic welfare, such as overcrowding, were not predictive.

There was a strong association between the husbands' alcohol consumption and domestic violence (p=0.001), as well as a high risk of possible caseness for women who were victims of domestic violence (OR=2.9, CI=1.2–6.8). Forty-six per cent of women reported being victims of domestic violence.

The prevalence of possible common mental disorders was determined using a cut-off point of 9 out of 20 for probable diagnoses. This threshold has been found to yield the best combination of sensitivity and specificity in studies comparing the instrument with clinical research diagnostic interview.^9^ Husbands' alcohol consumption was linked to domestic violence and there was a high risk for possible caseness in women who were the victims of domestic violence. As the age increased, so did the caseness (Table 2).

Having a concurrent physical illness or chronic pain increased the risk of being a case (OR=4.5, CI=1.8–11). As would be expected, there was an association between age and physical illness (*t*=12.938, df=138; p=0.000).

Among cases, 34% were on medication compared with 12% of non-cases (p=0.021). Since the SRQ comprises questions relating to somatic symptoms, it could be that caseness as defined by the SRQ picked up physical illness as well as psychosomatic symptoms. To elucidate this, a score was calculated for the SRQ questions relating only to psychological distress. The fact that physical illness was still associated with psychological distress (*t*=0.697, df=133, p=0.001) confirms the previous results regarding the high sensitivity of the SRQ (Table 3).

**Table 3:** Association between physical illness and mean SRQ score for psychological symptoms (0–10)

	Physical illness	No physical illness	*t* test F (p value)	Significance (2-tailed)
Mean score (±SD)	7.274 (3.11)	5.338 (3.27)	0.697 (0.405)	0.001

Of the women, 50% reported that they had at some time thought of ending their life, particularly those who were older (Mann–Whitney Z= −3.928, p=0.000) widows, and those with high SRQ scores (Mann–Whitney Z= −7.764, p=0.000).

Having a physical illness and 2 or more children predicted suicidal ideation (Tables 4 and 5).

**Table 4:** Univariate factors associated with suicidal thoughts (chi-square test)

Suicidal thoughts	Yes *n* (%)	No *n* (%)	Chi-square p	Odds ratio
Widow	25 (13)	8 (4)	0.017	4 (1.2–13.3)
Physical illness or pain	68 (34)	41 (21)	0.007	3 (1.3–6.8)
Physical illness of family member	20 (10)	39 (20)	0.030	2.6 (1.1–6.4)

**Table 5:** Univariate factors associated with suicidal thoughts (ttest, Mann–Whitney)

Suicidal intent Mean (±SD)	Case	Non-case	*t* test F (p)	Significance (2-tailed)	Mann–Whitney Z (p)
Age	42	32.6	9 (0.003)	0.000	–3.928 (0.000)
Number of children	2.91	2.39	0.312 (0.577)	0.037	–1.924 (0.054)
SRQ total score	15.2	7.9	0.036 (0.849)	0.000	–7.764 (0.000)
SRQ psych score	9.3	4.2	0.312 (0.577)	0.000	–8.771 (0.000)

## DISCUSSION

Before discussing the findings, some serious limitations of the present study must be discussed. First, only a screen was used and clinical structured examination was not conducted. Thus, it is possible that there may be more false-positive results and, second, as the sample was not random but purposive, the findings may not be generalizeable. The sample had only women, thus any comparisons across gender in other studies and other countries may not be valid. The sample also included more unemployed people reflecting a selection bias. Furthermore, the number of subjects was relatively small; hence, the results must be seen as tentative.

This study observed a higher than expected frequency of possible common mental disorders. Studies from India have previously reported rates of 18%–40%.^8^,^10^ A high cut-off of 9 on the SRQ must mean that the rates are generally higher. This may indicate that the rates are genuinely high which may reflect the rural setting and poverty of the sample. As we did not use clinical interviews to confirm the diagnosis it is difficult to be certain about diagnosis. Ideally, a two-stage process should be conducted. These high rates also support the role of gender in common mental disorders.^11^ This is also in accord with studies from both developing and developed countries.^12^,^13^ This gender role may also be reflected in high levels of suicidal ideation. South Asian females in the West have been shown to have high rates of attempted suicide^14^,^15^ although from India studies have shown that it is the males who are ideators and completers.^16^ The link between physical illness and depression is not entirely surprising.

Coincidentally, we found a clear link between poverty and possible caseness, although poverty, unemployment and homelessness have been associated with depression.^17^ Physical illness, pain, domestic violence and lack of income along with more than two children at home increased the risk of caseness. Interestingly, adverse life events did not seem to influence the risk of caseness. The features of physical illness, pain, domestic violence and husbands' alcohol consumption are likely to contribute to lack of income and increasing poverty, thereby making it possible that this may contribute directly to caseness or act as an intermediary. The risk of caseness relating to increasing age may also reflect the increase in physical illness and medication with increasing age. Again, whether this is related as an intermediary factor cannot be concluded from the present data.

Suicidal ideation over the lifetime was surprisingly high, in that nearly half of the sample had considered it and again age, number of children, being a widow and physical illness of a family member all seem to contribute to suicidal ideation. Alcohol consumption by the husband and domestic violence and confirmed the previous findings of Ponnudurai *et al*.^18^

In spite of the small numbers studied, poverty, domestic violence and physical illness were linked with depression in women. In spite of not having a control group, the high frequency of possible caseness on SRQ indicates the need for future detailed psychological assessments in the context of medical illness, especially chronic illness and pain. Gender role expectations must be explored to understand the pressures on women and their social and individual functioning.
